# The Effect of Socio-Economic Predictors of Chronic Diseases in Ghana: Results of a Nationwide Survey

**DOI:** 10.5539/gjhs.v5n5p115

**Published:** 2013-06-16

**Authors:** Bashiru I. I. Saeed, A. R. Abdul-Aziz, Samuel Nguah Blay, Xicang Zhao

**Affiliations:** 1 School of Finance and Economics, Jiangsu University, Jiangsu, China; 2 Mathematics and Statistics Department, Kumasi Polytechnic, Kumasi, Ghana; 3 Paediatric Department, Komfo Anokye Teaching Hospital, Kumasi, Ghana

**Keywords:** chronic disease, socio-economic predictors, health, Ghana

## Abstract

Socio-economic predictors of chronic diseases in Ghana are not well understood and their influence has been relatively overlooked. This paper seeks to examine the influence of socio-economic predictors of chronic diseases in Ghanaians three different age groups. The data employed in the study were drawn from Global Ageing and Adult Health survey conducted in Ghana by SAGE and was based on the design for the World Health Survey. The survey was conducted in 2007 and collected data on socio-economic characteristics and other variables of the individuals interviewed. The overall results suggest that chronic diseases in relatively older Ghanaians reflects social and economic exposures with the differentials observed only partially explained by current social and economic conditions. Our results were by and large very much expected from the current medical knowledge available.

## 1. Introduction

Good health does not only improve the welfare of the individual but also improve national output as well as economic growth. As mortals, sickness has become inevitable to every individual and can never be dealt with completely. Apart from other contributing factors (such as genetics), some of these sicknesses might be strongly influenced by the level of socio-economic attainment of individuals. The effect of socio-economic status on health condition has been studied extensively in both social and medical science literature (Feinstein, 1993; Tuinstral et al., 1998; Saastamoinen, Leino-Arjas, Laaksonen, & Lahelma, 2005; Case, Paxson, & Vogl, 2006; Verceli et al., 2006; Garemo, Lenner, Nilson, Borres, & Strandvik, 2007; Richter et al., 2009; Hallerod & Gustafsson, 2011). However, such related studies are limited as far as sub-Saharan African countries including Ghana are concerned. Understanding such a relationship has an important role to play in health policy design and implementation. According to Hallerod and Gustafsson (2011), if we think that income affects health and that a more equal income distribution will decrease inequity in health, then we need to pursue a policy that results in a more equal income distribution.

Most of the previous literature has been focused on socio-economic status and health conditions in general. The present research intends to determine the effect of socio-economic status on chronic illness. The importance of socio-economic status as a distinct channel in explaining the real effect of chronic illness has recently been given empirical support (Elliott, Smith, Penny, Smith, & Chambers, 1999; Hayward, Miles, Crimmins, & Yang, 2000; Walker, Peterson, & Millen, 2001; Paeratakul, Lovejoy, Ryan, & Bray, 2002; Eriksen, Jensen, Sjøgren, Ekholm, & Rasmussen, 2003). For instance, Laaksonen et al. (2005) argued that poor nutrition and inferior housing associated with low income levels contribute to infectious and chronic diseases, injuries, and poor development which create further barriers to children's success. Ballon et al. (2008) investigated the relationship between socioeconomic status and severity of chronic kidney disease in a retrospective, cross-sectional analysis involving patients at the Sheffield Kidney Institute, at United Kingdom. From their results, they concluded that low socioeconomic status is associated with the development and progression of chronic kidney disease as well as its severity. Saastamoinen et al. (2005) also studied socio-economic differences in the prevalence of acute, chronic and disabling chronic pain among ageing employees of the city of Helsinki. In their conclusion they reported that chronic pain was a prevalent problem among employees and showed that a clear socio-demographic and socio-economic differences.

Heiner et al. (2012) argued that the associations between the intake of vegetables and fruit and the risk of several chronic diseases show that a high daily intake of these foods promotes health. Socio-economic status, oral symptoms and compliance with dietary guidelines were associated with general health status (Brennan & Singh, 2012). Similarly, socio-economic status and oral health factors related to tooth loss and chewing ability have been related to compliance with dietary guidelines through food purchasing in older adults (Brennan & Singh, 2011). However, psycho-social factors have been suggested as important including sense of coherence (Lindmark, Stegmayr, Nilsson, Lindahl, & Johansson, 2005), food related self-efficacy and intentions (Gittelsohn et al., 2006), and identification with healthy eating (Strachan & Brawley, 2009).

The issue of the association between socio-economic status and chronic illness is of great importance to the economy of Ghana. For instance, if the effects of socioeconomic factors on economic output take place via changes in health condition (chronic illness), it is possible that a policy that results in equal distribution of socioeconomic wealth will have a positive effect. This paper aims to contribute to the existing literature on health related studies in Ghana by providing empirical results pertaining to the significant effect of socio-economic status on the presence or absence of chronic diseases in ageing adults population in Ghana. It is expected that the results would have implications for the successful implementation of health and socioeconomic policies.

## 2. Material and Methods

### 2.1 Sampling Procedures

The data employed in this study were drawn from the World Health Organisation’sGlobal Ageing and Adult Health (SAGE). This aims to address the gap in reliable data and scientific knowledge on ageing and health in low – and middle –income countries. SAGE is a longitudinal study with nationally representative samples of persons aged 50+ years in Ghana with a smaller sample of adults aged 18-49 years. Instruments are compatible with other large high-income country longitudinal ageing studies. Wave 1 was conducted during 2008-2009 and included a total of 4770 respondents aged 50+ and 803 aged 18-49.

Few low- and middle-income countries have data on levels and distribution of health and disability among the older population, much less on which morbidity trajectory their respective ageing populations are following: expansion of morbidity, where people are living longer with more disease and disability; compression of morbidity, where longevity increases but with delays in the age at onset and progression of disease; or a dynamic equilibrium, where longevity and disability rates both increase, but the severity of disability is not as severe. Large-scale longitudinal research in multiple settings is required to provide comparable information on health and well-being, as well as to track the impact of health interventions and policies within and across countries. The World Health Organization (WHO) Study on global AGEing and adult health (SAGE) aims to address this gap with a global perspective. Its core consists of national longitudinal studies of older people in lower- and upper-middle-income brackets, complemented by research collaborations with existing local population-based research studies, comparisons with other domestic ageing studies, and also comparisons to studies outside Ghana. SAGE is a longitudinal study with nationally representative samples of persons aged 50+ years in, Ghana, with comparison samples of younger adults aged 18–49 years in Ghana. The main aim is to generate valid, reliable and comparable information on a range of health and well-being outcomes of public health importance, in adult and older adult populations. The core SAGE countries provide a broad representation from different geographic regions of the world, different levels of economic development and different stages in the demographic and health transition, and include the two most populous countries of the world. SAGE is also designed to provide results that are comparable to ageing studies in high-income countries, such as the US Health and Retirement Study, the English Longitudinal Study on Ageing and the Collaborative Research on Ageing in Europe (COURAGE in Europe) Project in three countries. Data resource area and population coverage. Face-to-face interview was conducted in Ghana (2008–09). Multistage cluster sampling strategies were used where households were classified into one of two mutually exclusive categories:

(1) all persons aged 50 years and older were selected from households classified as ‘50+ households’; and
(2) one person aged 18–49 years was selected from a household classified as an ‘18–49 household’.

Household enumerations were carried out in the final sampling units. One household questionnaire was completed per household where a household informant and individual respondent need not be the same individual. One individual was selected from 18–49 households, whereas for 50+ households all individuals aged 50+ were invited to complete the individual interview. Proxy respondents were identified for selected individuals who were unable to complete the interview. Household-level analysis weights and person-level analysis weights were calculated for each country, which included sample selection and a post-stratification factor. Post stratification correction techniques used the most recent population estimates provided by the Ghana Statistical Service. The pooled Wave 1 national total for individual respondents included 4770 respondents aged 50+ and 803 aged 18–49.

A standardized survey instrument, set of methods, interviewer training and translation protocols are used in all SAGE countries. The SAGE household questionnaire consists of

(1) a household roster and modules about the dwelling, income, transfers in and out of the household, assets and expenditures;
(2) an individual questionnaire with modules on health and its determinants, disability, work history, risk factors, chronic conditions, care giving, subjective well-being, health care utilization and health system responsiveness;
(3) a proxy questionnaire about health, functioning, chronic conditions and health care utilization;
(4) a verbal autopsy module questionnaire to ascertain the probable cause of death for deaths in the household in the 24 months prior to interview or between interview waves; and
(5) appendices including Show cards to assist with the interviews. In addition, SAGE Wave 1 included anthropometric measurements (height, weight, waist and hip circumferences), blood pressure measures and a blood sample via finger prick, and performance tests including near and distant vision, a timed 4-m walk, grip strength, lung function and cognition.

International standards were used to harmonize education levels and occupations.

### 2.2 Model Specification

The independent variables considered in the study include gender, age, formal schooling, job employment, income level, ethnicity, height, marital status, religion and presence of obesity. For each chronic disease (i.e. the dependent variable) a model is specified to determine the presence or absence of the diseases based on the independent variables considered. In this study the chronic conditions considered include hypertension, diabetes, angina (chest pain), arthritis (joint pain), stroke, visual impairment and obesity. In the analysis, obesity was considered as a chronic disease as well as a risk factor for other chronic diseases.

Due to the dichotomous nature of the response variables: the presence or absence of any chronic disease, the appropriate choice of model considered for the analysis was Binary Logistic Regression (binary logit) model.

For the self reported health status, a model was specified to determine the presence or absence of any chronic disease given all the demographic and socio-economic characteristics available. The paper specified a binary logit model for each chronic disease considered.

## 3. Results

### 3.1 Data Description

Table 1 presents the preliminary statistics of the data used in this study. It represents the numerical strength and row percentage for characteristics of interest. Detail analysis followed so as to bring out the inferential part. The total number of observations for this study is 5573 though with some negligible missing observation.

**Table 1:** Sample characteristics

Variables	Frequency	(%)
**Chronic Disease**		
*Presence of Hypertension*	2829	56.1
No Hypertension	2214	43.9
*Presence of Diabetes*	177	3
No Diabetes	4913	97
*Presence of Angina (Chest Pain)*	149	2.9
No Angina (Chest Pain)	4939	97.1
*Presence of Arthritis (Joint Pain)*	580	11.4
No Arthritis(Joint Pain)	4509	88.6
*Presence of Stroke*	120	2.4
No Stroke	4970	97.6
*Presence of Visual Impairment*	1263	24.9
No Visual Impairment	3812	75.1
*Presence of Obesity*	500	9.1
No Obesity	4471	89.9
**Gender**		
Male	2816	50.6
Female	2749	49.4
**Age**		
Below 40	455	8.3
40 – 59	2267	40.7
60 and above	2841	51.0
**Marital Status**		
Not-married	2157	39.0
Married	3373	61.0
**Schooled**		
No	2576	50.5
Yes	2520	49.5
**Employer**		
Public	469	9.4
Private	207	4.1
Self-employed	3967	79.5
Informal	348	7.0
**Enough Money**		
Completely	71	1.3
Mostly	276	5.5
Moderately	1186	23.5
A little	2197	43.5
Not at all	1324	26.2
**Religion**		
Christianity	3546	70.0
Islam	797	15.7
Other	750	14.3
**Ethnicity**		
Akan	2465	49.1
Ewe	333	6.6
Ga-adangbe	528	10.5
Gruma	255	5.1
Grusi	48	1.0
Guan	82	1.6
Mande-busanga	82	1.6
Mole-dagbon	126	2.5
Other	1101	21.9

From the table 1, a little over half of the respondents suffered from ***Hypertension*** whiles a quarter suffered from ***Visual Imparment***. The rest were not very worrying as Ghanaians registered low percentages in each. There is fairly even distribution among ***males*** and ***females*** respectively. The strength of the age category groups tend to heavily favor the ***adults***, and particularly ***older adults*** respectively. Nearly two-thirds of the respondents are ***married***. There was no significant difference in the distribution between those who have ***ever schooled*** and those not. More than two-thirds of the respondents were ***self-employed*** with few of them being employed by ***private*** firms. A fairly distribution of a quarter of persons respectively have had ***moderate amount*** ***of money*** and ***not at all*** to meet their needs. Interestingly a little over two-fifth have had ***little*** for their needs. Half of the entire ethnic groups were Akans. More than two-thirds of the respondents belong to Christianity faith.

To determine the significant effect of socio-economic variables on chronic illness development, a binary logistic regression model was specified. In the fitted model each chronic disease considered, as dependent variable was treated as either present or absent.

Table 2 present the estimated odds ratio from the seven (7) logit models. Seven chronic diseases (Diabetes, Angina, Arthritis, Stroke, Visual Impairment and Obesity) were considered with the socio-economic variables being used as the explanatory variables in the model.

**Table 2:** Estimated odds ratio for chronic illness models

Variables	Hypertension	Diabetes	Angina (Chest Pain)	Arthritis (Joint Pain)	Stroke	Visual Impairment	Obesity
**Gender**							
Male	Ref	Ref	Ref	Ref	Ref	Ref	Ref
Female	1.014	1.507**	1.311	1.412***	0.638	1.036	2.972***
**Age**							
Below 40	Ref	Ref	Ref	Ref	Ref	Ref	Ref
40 – 59	2.199***	2.738**	3.821	3.972***	2.579	1.694***	1.365
60 and above	2.681***	5.880***	6.896**	9.791***	5.492**	3.863***	0.899
**Marital Status**							
Not-married	Ref	Ref	Ref	Ref	Ref	Ref	Ref
Married	0.792***	1.310	0.901	0.915	0.613	0.981	0.939
**Schooled**							
No	Ref	Ref	Ref	Ref	Ref	Ref	Ref
Yes	0.912	1.383	0.709	0.921	1.786**	0.679***	1.513***
**Employer**							
Public	Ref	Ref	Ref	Ref	Ref	Ref	Ref
Private	1.109	0.620	0.610	0.836	0.515	0.579**	1.085
Self-employed	0.718***	0.475***	0.968	0.674**	0.410**	0.958	0.952
Informal	0.775	0.286***	0.750	0.588	0.321**	0.729	1.059
**Enough Money**							
Completely	Ref	Ref	Ref	Ref	Ref	Ref	Ref
Mostly	0.772	1.671	0.377	1.582	0.330	0.909	1.799
Moderately	0.655	1.307	0.558	1.799	0.556	-0.96	1.336
A little	0.608	1.372	0.598	2.671	0.652	0.895	0.874
Not at all	0.522**	1.229	0.632	3.606**	---	1.374	0.623
**Religion**							
Christianity	Ref	Ref	Ref	Ref	Ref	Ref	Ref
Islam	1.325**	0.290***	0.328***	1.181	1.142	1.024	1.437
Other	0.898	0.306***	0.842	1.145	0.377	0.990	0.470***
**Ethnicity**							
Akan	Ref	Ref	Ref	Ref	Ref	Ref	Ref
Ewe	0.885	0.653	1.011	0.531**	1.352	1.037	1.763***
Ga-adangbe	0.862	1.044	1.724**	0.834	1.291	1.062	1.976***
Gruma	0.969	1.330	1.519	0.828	1.55	1.093	2.631***
Grusi	0.882	1.779	---	0.949	---	0.573	1.088
Guan	0.537**	1.816	1.184	0.735	0.586	0.706	0.911
Mande-busanga	0.893	0.418	0.544	1.324	1.256	1.857	1.757
Mole-dagbon	1.042	1.381	3.164**	1.019	1.293	0.872	0.824
Other	0.533***	0.724	0.723	0.341***	0.613	0.981	0.717
**Obese**							
No	Ref	Ref	Ref	Ref	Ref	Ref	---
Yes	2.204***	1.891***	1.670**	1.979***	1.283	1.036	---
**Model-fit Statistics**							
No. of observation	4662	4679	4633	4677	4570	4661	4679
LRT (*P-value*)	<0.001	<0.001	<0.001	<0.001	<0.001	<0.001	<0.001
AIC	6172.20	1362.28	1226.03	3091.79	887.54	4880.20	2829.53

** indicate 5% significance level respectively

*** indicate 1% significance level respectively

From the results shown in the table, Females are 2.97 times as likely to be obese as compared to their male counterparts. Also they are 51% more likely to be diabetic than their male counterparts. Female respondents are 1.4 times as likely to have joint pains as to their male counterparts. 51% of the educated are more likely to be obese as against their non-educated counterparts.

Adult Ghanaians are 2.738 times as likely to suffer from diabetes as to their young adults counterparts. It is much higher for older adults. There is also an indication that, adults and older adults are 2.199 and 2.681 times as likely to be hypertensive as to their young adults group.

Individuals who are 60 and above are almost seven times as likely to experience chest pain as to their counterparts who are less than 40 years. Ghanaian adults are almost four times as likely to have arthritis as to their young adult counterparts. Similarly, older adults are almost ten times as likely to have joint pains as to their young adults counterparts. Ghanaians who respectively in 40-59 years and 60 and above age bracket are respectively 1.7 and 3.9 times as likely to be visually impaired as to less than 40 years group. Older adult Ghanaians are 5.5 times as likely to get stroke as to young adults. Similarly, married persons are 0.792 times as likely to be hypertensive as those who are not. Educated Ghanaians are 79% more likely to have stroke as opposed to those who are not. Ghanaians who are educated are 32% less likely to be visually impaired as opposed to non-educated. Again, respondents who are self-employed are 0.718 times as likely to be hypertensive as to their counterparts in the public sector. Self-employed persons are 33% less likely to experience arthritis as compared to their counterparts in the public sector. Ghanaians who are self-employed are 52.5% less likely to be diabetic than their colleagues in the public sector. Also those in the informal sector are 71.4% far less likely to be diabetic than their counterparts in the public sector. Self-employed and those in the informal sector are respectively 0.4 and 0.3 times as likely to get stroke as to those in the public sector. Persons who are privately employed are 42% less likely to be visually impaired as opposed to their counterparts in the public sector.

Interestingly, those who do not have enough money are 0.522 times as likely to have hypertension as to their counterparts who have completely enough to take care of their health needs. Ghanaians who do not have money at all to meet their needs are 3.6 times as likely to experience arthritis as to those who completely have enough.

In another development, persons of Islamic faith are 1.323 times likely to suffer from hypertension as compared to their Christian counterparts. Interestingly persons of Christian faith are 71% more likely to be diabetic as their Islamic counterparts. The same (69.4%) can be said for the other religious faith. Ghanaians of Islamic faith who are numerically disadvantaged are 67% far less likely to have chest pains as compared to those of Christian faith. Other religious groups beside Islam are 53% less likely to be obese as against their Christian counterparts.

Respondents who are Guans and other tribes beside stated ones are highly significant. They are 0.537 and 0.533 times as likely to be hypertensive as to their Akan counterparts. Ghanaians of Ga-Adangbe extractions are 1.7 times as likely to have chest pains as to their Akan counterparts. Similarly, Mole-Dagbon are 3.2 times as likely to also have chest pains as to their Akan counterparts.

## 4. Discussion and Conclusion

This study confirmed the effect of socio-economic status on health condition that has been studied extensively in both social and medical science literature (Feinstein, 1993; Tuinstral, Groothoff, Van den Heuvel, & Post, 1998; Saastamoinen et al., 2005; Case et al., 2006; Verceli et al., 2006; Garemo et al., 2007; Richter et al., 2009; Hallerod & Gustafsson, 2011). More than 50% of the respondents suffered from ***Hypertension*** whiles Individuals who are 60 and above are very highly likely to experience chest pain. A fairly distribution of a quarter of persons respectively have had ***moderate amount*** ***of money*** and ***not at all*** to meet their needs. Again, respondents who have symptoms of obesity are 67% more likely to experience angina as opposed to those who have no symptom recorded

Direct comparison with chronic disease among the general population cannot be made, since suitable data are lacking. Nevertheless, we assume that due to “literacy effect” ie those Ghanaians who are educated are 32% less likely to be visually impaired as opposed to non-educated. This goes to support Ballon et al. (2008) investigation that established relationship between socioeconomic status and severity of chronic kidney disease in a retrospective, cross-sectional analysis involving patients at the Sheffield Kidney Institute, at United Kingdom. From their results, they concluded that low socioeconomic status is associated with the development and progression of chronic kidney disease as well as its severity. The importance of socio-economic status as a distinct channel in explaining the real effect of chronic illness has recently been given empirical support (Elliott et al., 1999; Hayward et al., 2000; Walker et al., 2001; Paeratakul et al., 2002; Eriksen et al., 2003). For instance, Laaksonen et al. (2005) argued that poor nutrition and inferior housing associated with low income levels contribute to infectious and chronic diseases.

Preventive health behaviors has rarely been used in studies of chronic disease. However, Heiner et al. (2012) argued that the associations between the intake of vegetables and fruit and the risk of several chronic diseases show that a high daily intake of these foods promotes health. While this study examined effect of socioeconomic predictors on chronic, an alternative approach is to examine multiple effects on health. Much in the same vein, socio-economic status, oral symptoms and compliance with dietary guidelines were associated with general health status (Brennan & Singh, 2012). Similarly, socio-economic status and oral health factors related to tooth loss and chewing ability have been related to compliance with dietary guidelines through food purchasing in older adults (Brennan & Singh, 2011). According to a study on psycho-social factors that have been suggested as important including sense of coherence (Lindmark et al., 2005), food related self-efficacy and intentions (Gittelsohn et al., 2006), and identification with healthy eating (Strachan & Brawley, 2009).

Our focus on current chronic disease may exclude some of the regularly recurrent ones, but nevertheless captures all constant chronic disease. This focus is likely to diminish recall bias, but it may have caused underestimation of the overall prevalence of chronic disease

In conclusion, chronic disease was a prevalent problem and associated with socioeconomic status among ageing Ghanaians. Older adults were at highest risk for chronic disease, but the level of risk was high also among adults. In similar fashion, Unmarried Ghanaians are also at high risk for chronic disease. Early risk from young adults due to chronic disease is a serious problem that needs further investigation. Those in formal educational groups, Islamic faith and female respondents were at a high risk for chronic disease. According to this study relatively, all categories of employment status are less likely to be affected by chronic disease. This suggests that prevention of chronic disease, in order to be effective, is needed already in the earlier stages of the growing individual and work career. The challenge of this study is the effect of unit and item non-respone which resulted in not achieving 100% response rate.

This paper has examined the problems of chronic diseases in Ghana in relation to socioeconomic predictors. The study established existing relationship between socioeconomic predictors and chronic diseases in Ghana. In particular, as respondent age increases, there is direct development and progression of chronic diseases as well as its severity. This goes to confirm the expectation from the current medical knowledge available.

In line with the analysis of the Data obtained (WHO Study on Global Ageing and Adult Health) and also other observations made in the course of this study, we make the following recommendations:

The socioeconomic predictors could be structured to take into account explicitly the underlying assumptions involved in minimizing chronic diseases. This could benefit both chronic disease patients and health practitioners concerned in dealing with the various health conditions. Further studies are needed to detect psychosocial and physical risk factors at work, associated with socio-economic effects in chronic disease.

## References

[ref1] Agresti A (2007). An Introduction to Categorical Data Analysis.

[ref2] Bello A. K, Peters J, Rigby J, Rahman A. A, El Nahas M (2008). Socioeconomic Status and Chronic Kidney Disease at Presentation to a Renal Service in the United Kingdom. Clinical Journal of the American Society Nephrology.

[ref3] Brennan D. S, Singh K. A (2012). Dietary, Self-Reported Oral Health and Socio-Demographic Predictors of General Health Status among Older Adults. The Journal of Nutrition, Health & Aging.

[ref4] Brennan D. S, Singh K. A (2011). Grocery purchasing among older adults by chewing ability, dietary knowledge and socio-economic status. Pub Health Nutr.

[ref5] Case A, Paxson C, Vogl T (2006). Socio-economic Status and Health in Childhood: A Comment on Chen, Martin, and Matthews (2006). NBER Working Paper vNo. 12267, National Bureau of Economic Research.

[ref6] Elliott A. M, Smith B. H, Penny K. I, Smith W. C, Chambers W. A (1999). The Epidemiology of Chronic Pain in the Community. Lancet.

[ref7] Eriksen J, Jensen K. M, Sjøgren P, Ekholm O, Rasmussen N. K (2003). Epidemiology of Chronic Non-malignant Pain in Denmark. Pain.

[ref8] Feinstein J. S (1993). The Relationship between Socioeconomic Status and Health: A Review of the Literature. The Milbank Quarterly.

[ref9] Garemo M, Lenner R. A, Nilson E. K, Borres M. P, Strandvik B (2007). Food Choice, Socio-economic Characteristics and Health in 4-year Olds in a Well-educated Urban Swedish Community. Clinical Nutrition.

[ref10] Gittelsohn J, Anliker J.A, Sharma S, Vastine A. E, Cabellero B, Ethelbah B (2006). Psychosocial determinants of food purchasing and preparation in American Indian households. J Nutr Educ Behav.

[ref11] Hallerod B, Gustafsson J.-E (2011). A Longitudinal Analysis of the Relationship between Changes in Socio-economic Status and Changes in Health. Social Science and Medicine.

[ref12] Hayward M. D, Miles T. P, Crimmins E. M, Yang Y (2000). The Significance of Socioeconomic Status in Explaining the Racial Gap in Chronic Health Conditions. American Sociological Review.

[ref13] Heiner B, Angela B, Achim B, Sabine E, Dirk H, Anja K, Bernhard W (2012). Critical review: vegetables and fruit in the prevention of chronic diseases. Eur J Nutr.

[ref14] Laaksonen M, Rahkonen O, Martikainen P, Lahelma E (2005). Socioeconomic Position and Self-Rated Health: The Contribution of Childhood Socioeconomic Circumstances. American Journal of Public Health.

[ref15] Lindmark U, Stegmayr B, Nilsson B, Lindahl B, Johansson I (2005). Food selection associated with sense of coherence in adults. Nutr J.

[ref16] Paeratakul S, Lovejoy J. C, Ryan D. H, Bray G. A (2002). The Relation of Gender, Race and Socioeconomic Status to Obesity and Obesity Comorbidities in a Sample of US Adults. International Journal of Obesity.

[ref17] Richter M, Erhart M, Vereecken C. A, Zambon A, Boyce W, Gabhainn S. N (2009). The Role of Behavioural Factors in Explaining Socio-economic Differences in Adolescent Health: A Multilevel Study in 33 Countries. Social Science and Medicine.

[ref18] Saastamoinen P, Leino-Arjas P, Laaksonen M, Lahelma E (2005). Socio-economic Differences in the Prevalence of Acute, Chronic and Disabling Chronic Pain among Ageing Employees. Pain.

[ref19] Strachan S. M, Brawley L. R (2009). Healthy-eater identity and self-efficacy predict healthy eating behavior: a prospective view. J Health Psychol.

[ref20] Tuinstral J, Groothoff J. W, Van den Heuvel W. J. A, Post D (1998). Socio-economic Differences in Health Risk Behavior in Adolescence: Do they Exist?. Social Science and Medicine.

[ref21] Vercelli M, Lillini R, Capocaccia R, Micheli A, Coebergh J. W, Quinn M, Martinez-Garcia C, The ELDCARE Working Group (2006). Cancer Survival in the Elderly: Effects of Socio-economic Factors and Health Care System Features (ELDCARE project). European Journal of Cancer.

[ref22] Walker C, Peterson C, Millen N (2001). Investigating Socio-economic Status and Chronic Illness: Directions and Dilemmas. TASA 2001 Conference, The University of Sydney.

